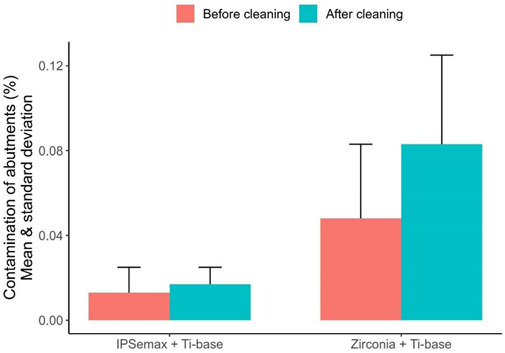# Qualitative and Semi-Quantitative Assessment of Processing-Related Surface Contamination of One- and Two-Piece CAD/CAM Abutments before and after Ultrasonic Cleaning

**DOI:** 10.3390/ma13143225

**Published:** 2020-07-20

**Authors:** Peter Gehrke, Cyrus Abazari, Kai Schlichter, Carsten Fischer, Dirk Duddeck, Georgios E. Romanos, Paul Weigl

**Affiliations:** 1 Department of Postgraduate Education, Master of Oral Implantology, Center for Dentistry and Oral Medicine (Carolinum), Johann Wolfgang Goethe University, Theodor-Stern-Kai 7, 60596 Frankfurt, Germany; 2 Private Practice, Bismarckstrasse 27, 67059 Ludwigshafen, Germany; 3 Private Practice, Karl-Berner-Straße 23, 79400 Kandern, Germany; cyrusabazari@web.de; 4 Private Practice, Konrad-Lerch-Ring 9, 76877 Offenbach, Germany; k.schlichter@dr-werling.de; 5 Sirius Ceramics, Lyoner Str. 44, 60528 Frankfurt, Germany; fischer@sirius-ceramics.com; 6 Medical Materials Research Institute, Max-Planck-Strasse 3, 12489 Berlin, Germany; duddeck@cleanimplant.com; 7 Department of Oral Surgery and Implant Dentistry, Center for Dentistry and Oral Medicine (Carolinum), Johann Wolfgang Goethe University, Theodor-Stern-Kai 7, 60596 Frankfurt, Germany; georgios.romanos@stonybrook.edu; 8 Department of Periodontology, School of Dental Medicine, Stony Brook University, Stony Brook, NY 11794, USA; 9 Department of Prosthodontics and Head of Department of Postgraduate Education, Master of Oral Implantology, Center for Dentistry and Oral Medicine (Carolinum), Johann Wolfgang Goethe University, Theodor-Stern-Kai 7, 60596 Frankfurt, Germany; weigl@em.uni-frankfurt.de

**Keywords:** CAD/CAM, contamination, debris, hybrid abutment, monotype abutment, planimetric measurement, ultrasonic cleaning, Finevo

## Abstract

Manufacturing processes of custom implant abutments may contaminate their surfaces with micro wear deposits and generic pollutants. Such particulate debris, if not removed, might be detrimental and provoke inflammatory reactions in peri-implant tissues. Although regulatory guidelines for adequate cleaning, disinfection, or sterilization exist, there does not appear to be a consistent application and data on the amount and extent of such contaminants is lacking. The aim of the present in vitro study was to evaluate the quality and quantity of processing-related surface contamination of computer-aided design/computer-aided manufacturing (CAD/CAM) abutments in the state of delivery and after ultrasonic cleaning. A total of 28 CAD/CAM monotype and hybrid abutments were cleaned and disinfected applying a three-stage ultrasonic protocol (Finevo protocol). Before and after cleaning, the chemical composition and the contamination of the abutments were assessed using scanning electron microscopy (SEM), dispersive X-ray spectroscopy (EDX), and computer-aided planimetric measurement (CAPM). In the delivery condition, monotype abutments showed a significantly higher amount of debris compared to hybrid abutments (4.86 ± 6.10% vs. 0.03 ± 0.03%, *p* < 0.001). The polishing process applied in the laboratory after bonding the hybrid abutment components reduces the surface roughness and thus contributes substantially to their purity. The extent of contamination caused by computer-aided manufacturing of custom abutments can be substantially minimized using a three-stage ultrasonic protocol.

## 1. Introduction

The continuous advancement in computer technology and material processing provides new opportunities in the field of implant restorative dentistry. Apart from conventional laboratory procedures, computer-aided design/computer-aided manufacturing (CAD/CAM) solutions are available for the production of custom implant abutments and fixed implant-supported single crowns or fixed partial prosthesis (FDPs) [1]. Traditionally, metal abutments and porcelain-fused to metal (PFM) crowns have been considered to be the gold standard, ensuring high survival rates of the restorations [2]. Titanium has been a reliable option for stock abutments in the posterior region because of its favorable mechanical properties [3,4]. As a result of esthetic reasons, there is an increasing trend to substitute titanium abutments with all-ceramic materials, i.e., alumina (Al_2_O_3_) and zirconia (ZrO_2_), to avoid peri-implant soft tissue discoloration in patients with a thin gingival biotype [5]. In addition to prefabricated stock abutments of different materials, custom CAD/CAM generated abutments can be engineered to re-establish the required emergence contour and supporting crown alignment, thus allowing the development of an anatomically oriented treatment result [6,7,8,9]. Currently, a range of ceramic CAD/CAM abutments with different geometries of the implant-abutment connection is available for different implant systems. An internal connection of a ceramic abutment can be obtained by means of: (1) a one-piece zirconia or alumina abutment (monotype abutment) with a cemented crown, (2) a directly veneered one-piece zirconia abutment (crown abutment), or (3) by the use of a CAD/CAM zirconia or lithium disilicate coping, which can be cemented on a titanium base (hybrid abutment). Hybrid abutments offer several potential benefits, such as favorable mechanical characteristics, simplicity and time efficiency by using a digital workflow [7,10,11,12].

An abutment is an integral component of the prosthetic restoration and is therefore in close contact with the surrounding peri-implant tissues. Abutment shape, its surface, and biocompatibility determine to a large extent the quality of the mucosal seal around an implant, preventing bacterial penetration and subsequent inflammation [13,14]. In addition, clinical investigations demonstrate the biological role of an abutment and the influence of the abutment height on marginal bone loss (MBL) and interproximal bone loss (IBL). The results suggest that the shorter the abutment height, the greater the marginal bone loss in cement-retained restorations. Thus, the abutment height seems to play a key factor in MBL and IBL confirming the desirability of maximizing the distance between crown and bone to obtain a more predictable bone preservation outcome [15,16,17]. CAD/CAM manufacturing processes for custom abutments be subject to potential surface contamination from micro-wear particles, cooling lubricants and general laboratory contaminants [18,19]. Debris have been found on the external and internal surfaces of custom implant abutments after laboratory procedures. Such debris, present at the critical implant-tissue interface, could trigger an inflammatory response of peri-implant tissues [20,21,22,23]. From a mechanical point of view, micro residues at the basal platform of an abutment could negatively impair the stability of the implant–abutment connection [24]. For this reason, cleaning and subsequent sterilization or high-level disinfection of the abutment surface is essential. European health regulations (International Organization for Standardization. EU/ISO 17664:2004: Sterilization of medical devices) and the guidelines of the American Dental Association (ADA) have approved cleaning and disinfection procedures for semi critical medical devices, such as CAD/CAM implant abutments [25]. They consider either sterilization by dry heat, steam treatment of the components under pressure at 134 °C (autoclaving), or an ultrasonic cleaning with approved disinfectants. Heat stable metal abutments can be safely autoclaved without affecting their material properties. However, vapor at such high temperature and pressure may damage macroscopically or microscopically abutment materials that are sensitive to heat, such as ceramic, resin, or the adhesive joint of hybrid abutments [26,27]. Even if sterilization aims to eliminate microbial contamination to achieve aseptic and sterile settings, debris from CAD/CAM abutments that arise during laboratory processing cannot be removed by a sterilization process alone. As an alternative, a three-stage ultrasonic cleaning process was introduced, which can be used universally in the dental laboratory and in the clinic to minimize contamination of CAD/CAM abutments [28]. The ultra-high frequency waves in combination with a disinfectant have been shown to mechanically and chemically remove contaminants from surfaces [16]. In vitro results confirm a generally good cell viability on titanium samples [29]. Enhanced cell attachment and reduced inflammatory response of human gingival fibroblasts (HGFs) have been shown to zirconia surfaces for this cleaning and disinfecting method [30].

In addition to overdue controlled clinical trials, efforts to ascertain the actual amount and extent of debris on customized abutments could be an important parameter to determine clinical relevance. Although the qualitative presence of organic and inorganic contaminants on CAD/CAM generated surfaces has been verified by microscopic and chemical analyzes [28,30], data on the quantity and scale of such contaminants are missing. Thus, the aim of the present study was to evaluate the quantity of processing-related surface contamination of CAD/CAM abutments before and after ultrasonic cleaning by means of scanning electron microscopy (SEM) and computer-aided planimetric measurement (CAPM). Prior to the cleaning process, a chemical characterization of the particle deposits was carried out by energy dispersive X-ray spectroscopy (EDX). The null hypothesis tested was that the planimetric analysis of SEM images is suitable for determining the projected surface of contamination and that the material and manufacturing process of the respective CAD/CAM abutment has no influence on the detected amount of contamination.

## 2. Materials and Methods

### 2.1. Study Design and Specimen Preparation

**Scheme 1:**
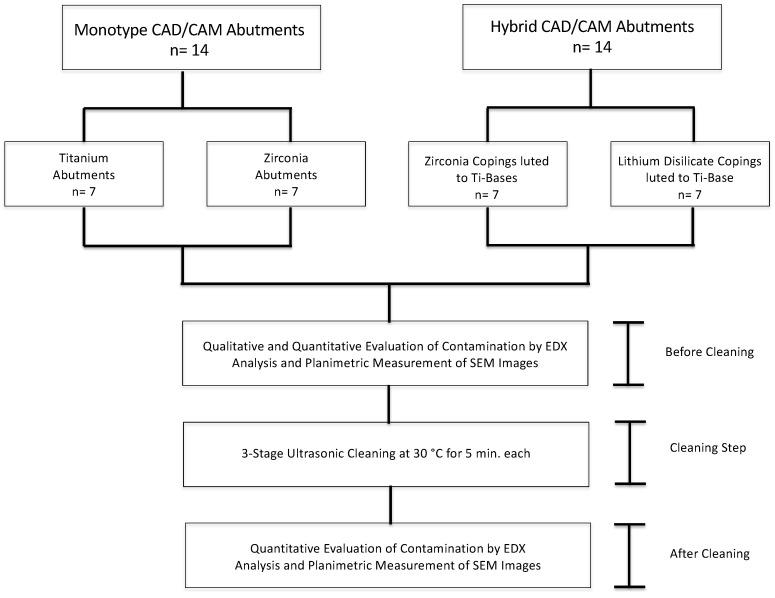
Study design.

**Figure 1:**
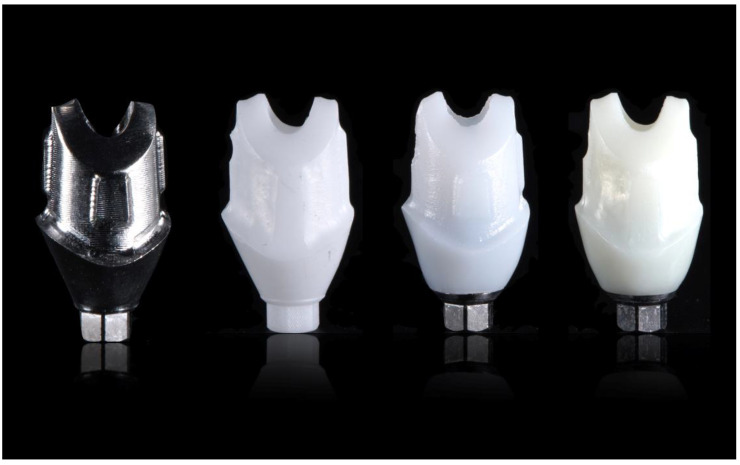
Examples of examined computer-aided design/computer-aided manufacturing (CAD/CAM) abutment specimens from left to right: titanium monotype abutment (CADAbut F, CAD Titanium), zirconia monotype abutment (CADAbut F, BeCe CAD Zirconia XH), zirconia hybrid abutment (CADAbut D, Zirconia LT CAD on Ti-Base), lithium disilicate hybrid abutment (CADAbut D, IPS e.max CAD LT on Ti-Base).

A total of 28 CAD/CAM abutments with a convex emergence profile were virtually designed (Implant Studio, 3Shape, Copenhagen, Denmark) and manufactured (CADAbut F and CADAbut D, BEGO Implant Systems GmbH & Co. KG, Bremen, Germany). The master cast of a clinical case in which the right maxillary central incisor had been replaced by an implant restoration served as the origin of the virtual abutment design. The case was restored using a regular platform two-piece implant with an internal taper connection and internal hex anti-rotation protection (Semados SCX D 4.1/L 11.5, BEGO Implant Systems GmbH & Co. KG, Bremen, Germany). The emergence profile of the peri-implant mucosa had been pre-conditioned by means of a temporary implant-supported single crown. The virtual design for all CAD/CAM abutments was identical in their outer geometry with a height of 10.5 mm and an abutment shoulder width of 6.5 mm. The main group of abutment specimens were assigned to two groups (n = 14 each) and further divided into two sub-groups (n = 7 each) according to the material and the CAD/CAM manufacturing process utilized: monotype abutments (one-piece,) and hybrid abutments (two-piece) (Scheme 1). CAM materials processed for monotype abutments included zirconia and titanium, while hybrid abutments consisted of zirconia or lithium disilicate copings cemented to prefabricated titanium bases in the laboratory (Ti-Base D 4.1 mm, Semados SCX, BEGO Implant Systems GmbH & Co. KG, Bremen, Germany) (Figure 1). A list of materials and manufacturers is shown in Table 1. In case of the two-piece hybrid abutments, the bonding surfaces of the titanium inserts and the ceramic copings were blasted (aluminum oxide particles 50 μm; 2 bar/0.25 MPa; 10 s; distance 10 mm) and cleansed with ethanol in the laboratory. Subsequently, the titanium inserts were wetted with a metal-primer (GC Metal Primer II, GC EUROPE N.V, Leuven, Belgium), whereas a bonding material was applied on the base of the ceramic copings (Monobond Plus, Ivoclar Vivadent, Schaan, Liechtenstein). All hybrid abutments were luted with a dimethacrylate/hydroxyethylmethacrylate-based cement (DMA/HEMA) (Multilink Implant, Ivoclar Vivadent, Schaan, Liechtenstein) according to the manufacturer’s recommendations. Finally, removal of the bonding excess was performed as well as polishing of the bonding joint with silicone polishers and polishing paste according to a previously documented protocol [30].

### 2.2. Cleaning of Abutment Samples

In accordance with current health regulations, the abutment samples were cleaned three times in an ultrasonic bath (Finevo Cleaning System, Bredent GmbH & Co. KG, Senden, Germany) at 30 °C for 5 min each, as previously described by the authors [28,30]. The first bath contained an antibacterial cleansing solution, the second bath contained 80% ethyl alcohol, and the third bath contained medically pure water (aqua dest.). After cleaning, the samples were kept in non-contact containers.

### 2.3. Qualitative Evaluation of Contamination

**Figure 2:**
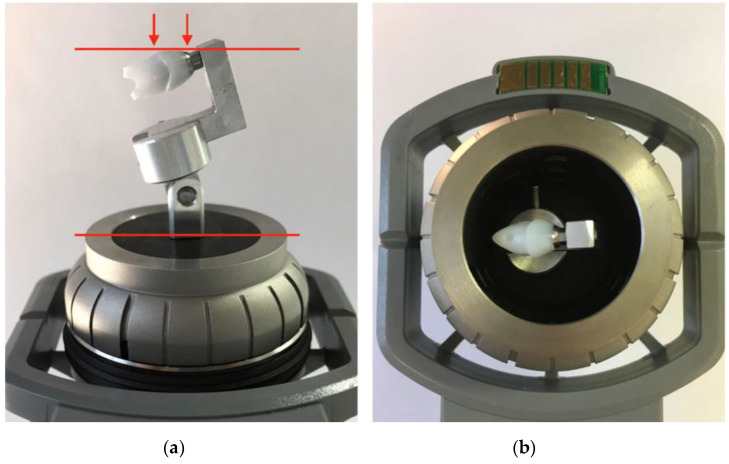
Test set-up: (**a**) Customized device and tilting turntable for exactly reproducible positioning of specimens during SEM; (**b**) specimen after lowering to optimum working distance.

In order to avoid artifacts, the samples were removed from their transport containers and transferred to a sterile work bench, followed by the SEM scan and EDX analysis process under clean room conditions (Laminar FlowBox, clean room class 5, DIN EN ISO 14644-1). The specimens were mounted on a fixture especially made for this project (Figure 2). This device ensured an exactly reproducible positioning of the specimen in order to examine the same area of the abutment shoulder before and after the cleaning process. With the aid of marking, the respective abutment was fixed into the specifically milled connection geometry (internal hexagon analogue to implant connection) of the custom-made device using the abutment retaining screw. The device was then mounted on a freely tilting turntable of a charge reduction sample holder (Phenom-World B.V., Eindhoven, The Netherlands) and finally lowered within the SEM unit for an optimum working distance of 6 mm. The sample holder is designed to reduce sample charging and to eliminate extra sample preparation, such as sputter coating. Non-conductive materials, as ceramic abutments can be imaged in their natural state providing unedited back scatter material contrast information. A tilt adjustment achieved a horizontal alignment of the specific abutment shoulder area to be examined. In order to chemically characterize the contamination, SEM and EDX were carried out at 5000× magnification for each abutment prior to the cleaning process.

### 2.4. Semi-Quantitative Evaluation of Contamination

**Figure 3:**
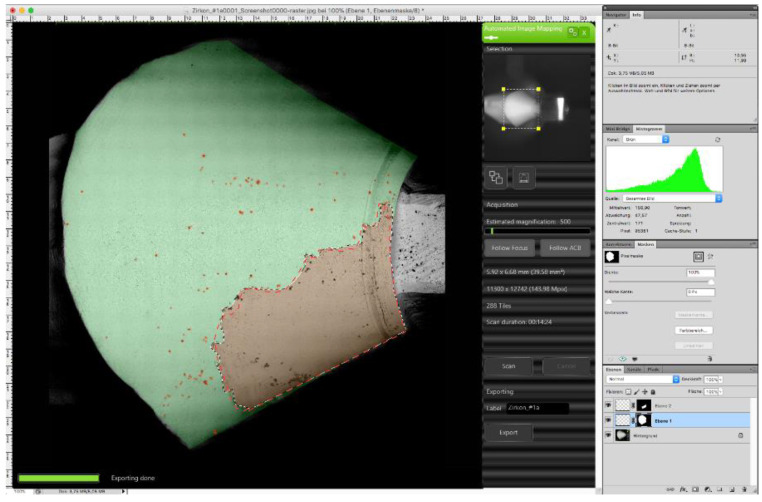
Computerized planimetric calculation of the proportion between area covered with contaminants (red) and the total surface of the abutment shoulder displayed (green).

Before and after ultrasonic cleaning of all abutments, a computer-assisted planimetric measurement (CAPM) of images from SEM was used to quantitatively examine projected surface contamination, considering a mathematical ratio calculation between the surface area covered with contaminants and the surface area of the abutment shoulder shown. This computerized method was previously applied to estimate plaque accumulation on tooth surfaces [31] and later to calculate undetected cement residues on implant abutments in experimental and clinical studies [32,33,34]. SEM images of the abutment shoulders at a standard 500x magnification were imported and analyzed with Adobe Photoshop (Adobe Systems Ltd., Vers.19.1.3 Europe, Uxbridge, UK). For each image obtained, the entire circumference of the projected shoulder was marked by hand using a free line tool (magnetic lasso-tool) of the software. Although the latter is manually guided, it is an automatically supported selection mechanism that can select an object, such as debris on a surface. The number of pixels was recorded algorithmically using the histogram option (Figure 3). The same procedure was applied to the contaminated area that followed the contours of the particles. The ratio between the projected area covered with debris and the shoulder area of the sample was calculated. All measurements were obtained by a single calibrated examiner (PG). Calibration was tested by double analysis of standardized digital SEM images from 10 selected abutment surfaces, with a one-week interval. The agreement coefficient was of 0.81, with a mean difference of 0.09 ± 0.83 (values ≥ 0.75 are considered excellent).

### 2.5. Statistical Analysis

Statistical analyses were carried out using the program packages STATISTICA (STATSOFT, Tulsa, OK, USA, 2010, Version 9.1) and BiAS (Epsilon-Verlag, Frankfurt, Germany, Version 11.02). Frequency distributions were used to characterize categorical variables. Descriptive statistics robust summaries (mean, standard deviation, median, minimum and maximum, and interquartile range) were used for data report. Mann–Whitney tests and Wilcoxon rank sum tests were used to compare independent groups for continuous variables. Significance was set at *p* < 0.05.

## 3. Results

**Figure 4:**
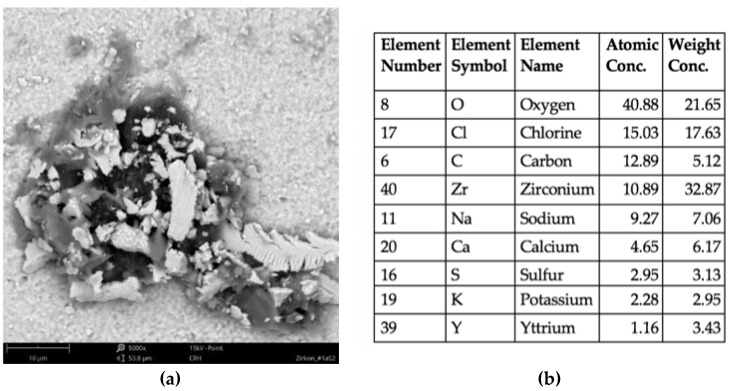
(**a**) SEM of debris on ZrO_2_ monotype specimen #1a before cleaning (5000×); (**b**) energy dispersive X-ray spectroscopy (EDX) analysis of respective SEM and chemical elements of contamination.

Prior to ultrasonic cleaning, the EDX analyses detected particles, deposits, organic and inorganic contamination to varying degrees on all CAD/CAM abutments tested (Example Figure 4a,b). The chemical elements of the identified particulate matter are listed separately for monotype and hybrid abutments and its sub-group in the Table 2, Table 3, Table 4 and Table 5.

### 3.1. Qualitative Evaluation of Contamination

Chemical analysis (EDX) of monotype and hybrid abutments revealed organic and inorganic contaminants prior to ultrasonic cleaning. Elements, atomic and weight concentration are shown in Table 2, Table 3, Table 4 and Table 5.

### 3.2. Semi-Quantitative Evaluation of Contamination

**Figure 5:**
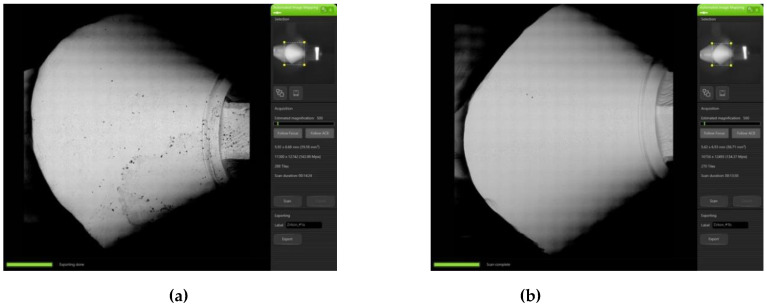
Representative examples of SEM images (500×) of zirconia monotype abutment #1a before (**a**) and after cleaning (**b**), showing the effective reduction of projected surface contamination.

The three-stage ultrasonic cleaning process generally reduced the surface contamination of all tested abutment specimens (Table 6 and Figure 5a,b). The mean value of surface contamination was lowered from 2.4% before to 0.1% after cleaning. However, it should be noted that the value for the standard deviation (SD) prior to cleaning were considerably higher (4.89%) as the corresponding mean value and thus significantly higher than the SD value after cleaning (0.12%).

#### 3.2.1. Comparison between Monotype and Hybrid Abutments CAD/CAM Abutments

In the delivery condition (before cleaning), all tested monotype CAD/CAM abutments showed a significantly higher amount of production-induced contamination compared to the hybrid abutments (4.86 ± 6.10% vs. 0.03 ± 0.03%, *p* < 0.001) (Table 7). The difference in deposits before and after ultrasonic cleaning for the two groups was therefore statistically considerably different. Importantly, the values for the standard deviations of the detected contaminations on monotype abutments were thus multiple times higher than on hybrid abutments.

#### 3.2.2. Monotype CAD/CAM Abutments

The mean value for the scanned contamination of titanium monotype abutments for the time “before cleaning” was 4.02 ± 2.61% and 5.70 ± 8.49% for the zirconia monotype abutments (Table 8). Thus, the standard deviation value (SD) of identified contaminants on titanium abutments was significantly lower than on zirconia abutments before ultrasonic treatment. In some extreme cases, the maximum contamination level of one aspect of the zirconia abutment shoulders amounted to 25% upon delivery. After ultrasonic cleaning, only 0.16 ± 0.11% of the examined pixels on titanium monotype abutments and 0.22 ± 0.16% on the zirconia monotype abutments were classified as contaminated. The percentage of surface contamination thus decreased by an average of 91.2% for all monotype abutments tested. This decrease displayed low SD values and was statistically significant for both titanium and zirconia abutments with *p* = 0.02.

#### 3.2.3. Hybrid CAD/CAM Abutments

In contrast, the average quantity of scanned contamination of the hybrid abutments IPSemax/Ti-Base and zirconia/Ti-Base before cleaning was only 0.01 ± 0.01% and 0.05 ± 0.034%, respectively (Table 9. After ultrasonic cleaning, 0.02 ± 0.01% and 0.08 ± 0.04% of the pixels examined were classified, as “contaminant”. This development was not statistically significant and demonstrated relatively small standard deviations from the mean for both hybrid abutment types. (*p* = 0.39 and *p* = 0.24).

## 4. Discussion

A range of custom CAD/CAM abutments is available in various materials and geometries. In contrast to stock abutments, they can be designed to restore the required emergence contour and supportive crown alignment, thus enabling the development of anatomically oriented treatment results. Due to its favorable mechanical properties, titanium has proven to be a clinically reliable material option for stock and custom monotype abutments in the posterior region [3,4]. To avoid peri-implant soft tissue discoloration in patients with a thin gingival biotype, monotype zirconia CAD/CAM abutments have been promoted to achieve better mucogingival esthetics [5]. A drawback of zirconia monotype abutments is their low fatigue/fracture resistance and wear at the implant interface following loading [7]. Recently introduced hybrid abutments consist of a prefabricated insert base of titanium onto which a customized CAD/CAM zirconia- or lithium disilicate coping is cemented in the laboratory. CAD/CAM hybrid abutments are clinically advantageous in highly stressed areas such as premolars and molars because of their excellent durability and pleasing esthetic results [10,11].

Manufacturing processes of custom titanium and zirconia implant abutments may contaminate the abutment surfaces with micro wear deposits, generic pollutants and laboratory consumables [18,20]. The qualitative presence of both organic and inorganic contaminants detected in the present survey (Table 2, Table 3, Table 4 and Table 5) confirms previous results of microscopic and chemical analyses [28,30]. Furthermore, elements of sulfur, phosphorus, and silicium could be detected. These acids and semi-metals were already correlated to CAD/CAM implant manufacturing processes in earlier studies and can be assigned to cleaning attempts after industrial milling [35]. Such particulate debris, if not removed, might act detrimental and provoke inflammatory reactions in peri-implant tissues [21,22,23,36]. Although regulatory guidelines for adequate cleaning, disinfection or sterilization exist, there does not appear to be a consistent application and data on the amount and extent of such contaminants is lacking [37]. The primary objective of the current in vitro study was therefore to evaluate the quantity of processing-related surface contamination on CAD/CAM abutments before and after ultrasonic cleaning using SEM, EDX, and CAPM. The results of the present planimetric evaluation conclude that ultrasonic treatment has the potential to effectively reduce the surface contamination on custom abutments. The first part of the null hypothesis, stating that computerized planimetry is suitable to determine the projected surface of contamination, could therefore be considered as accepted. As the degree of the cleaning result was strongly dependent on the individual fabrication procedure of the respective abutment, the second part of the hypothesis, which presupposes independence from the manufacturing process and contamination quantity, could consequently be rejected. On delivery, the monotype CAD/CAM abutments milled in a CAD/CAM center of an implant manufacturer exhibited a significantly higher degree of production-related contamination than the hybrid abutments produced in a dental laboratory. The mean projected proportion of contamination for all monotype abutments was 4 to 6% for one side of the abutment shoulder, with rare peak values of up to 25%. In contrast, the hybrid abutments showed comparatively low surface residues of 0.2% even before the actual cleaning protocol. Given this differing baseline, it is noteworthy that the residues on titanium and zirconia monotype abutments could be reduced up to 90% by sonication treatment (change in total contamination, Table 8. A reasonable explanation for the low contamination of hybrid abutments could be based on the manufacturing protocol of these two-piece CAD/CAM abutments. After the CAD/CAM-supported fabrication of corresponding zirconia or lithium disilicate copings, these ceramic copings are bonded to prefabricated titanium bases and then polished manually by the dental technician using silicone polishers of decreasing roughness. This polishing procedure reduces the surface roughness [30] and thus appears to make a decisive contribution to cleanliness and purity in the fabrication of hybrid abutments. In contrast, the tested monotype abutments were completely produced in a central production facility of an implant manufacturer, where typically no surface treatment such as polishing is performed. The apparent increase in the amount of debris on the hybrid abutment specimens (IPSemax on Ti-base, zirconia on Ti-base) after ultrasonic cleaning (0.02 ± 0.01% and 0.08 ± 0.04%, Table 9) remained at an extremely low level and can be attributed to standard deviations within the range of the planimetric measurement accuracy. The applied two-dimensional analysis (CAPM) in combination with x-ray spectroscopy (EDX) seems to suffer from limitations regarding its surface sensitivity, as minor quantities of contaminants may not be adequately recorded. Electron spectroscopy for chemical analysis (ESCA) and advanced three-dimensional measurements of technical cleanliness and foreign object analysis could provide clarification of these conflicting results. Another explanation for the apparent increase in contamination of hybrid abutments after ultrasonic cleaning could be that minimal residues of the silicone/rubber polishers used in the laboratory cannot be completely removed by ultrasound. However, the exact impact of the abutment material and post processing measures, such as polishing the surface, on the cleanliness of the abutment before and after ultrasonic cleaning still needs to be determined.

Although the reproducibility of the area to be scanned was ensured by employing a custom-device fixture, the limitation of the measuring area to only one side of the abutment shoulder could be regarded as a drawback of the present study. A further limitation relates to the relatively small number of samples per subgroup, which should be expanded by a larger number of samples in future investigations. This limitation was justified by the two-dimensional methodology of the computer-assisted planimetric measurement (CAPM). Since abutments are cylindrical objects and are configured to be rounded at their borders, this method cannot display the entire circumferential abutment surface. A more comprehensive three-dimensional surface measurement of cylindrical bodies might be achieved by means of specific digital microscopy, which, however, is rarely available in the research domain of implant dentistry due to the high cost investment. A 3-D evaluation could possibly detect higher residue levels and thus even increase the contamination results found.

The impact of different cleaning regimes has been disputed [38,39,40,41] and a sound clinical correlation between abutment cleanliness and peri-implant bone maintenance is yet to be demonstrated [42]. The current state of data regarding the influence of different cleaning procedures on the mechanical stability of hybrid abutments is controversial. While some in vitro studies concluded that both autoclaving and/or three-stage ultrasonic cleaning have no effect on the bond strength between zirconia copings and titanium bases [43,44,45], others showed a negative effect on the retentive strength and an increase in roughness of the resin-cement interface with a reduction of the mechanical stability of the hybrid construction [46,47]. However, it is important to point out that different metal primers and a varying number of thermal cycles were used in these studies. In addition, the type of cement, the mechanical and chemical modification of the surfaces, the geometry of the abutment and the luting gap can significantly influence the retention between the hybrid components, thus explaining supposedly contradictory results. Considering that hybrid abutments have not demonstrated a significant cleaning effect using the applied ultrasonic protocol, the risk of potential weakening of the adhesive bond should be avoided. Until this fact is conclusively assessed by predefined in vitro and in vivo analyses, the use of a three-step ultrasonic protocol can therefore only be recommended with limitations. Despite the fact that it is frequently used in daily routine, pure steam cleaning with a vapor device in the laboratory is not an approved cleaning and disinfection method. Whereas in vivo and in vitro investigations indicate that plasma pretreatment may be beneficial for the cleanliness of abutments [20,21,48], a more recent attempt by Farronato et al. concluded that plasma processing could not be effective enough [49]. As the current health regulations for the hygiene of semi-critical medical devices such as abutments only consider sterilization by dry heat, autoclaving or ultrasonic cleaning with approved disinfectants, it should be emphasized that plasma treatment is not a validated cleaning modality. The decision whether to ultrasonically clean and disinfect or sterilize a CAD/CAM abutment should be determined both by the applicable hygiene regulations and by the type of abutment material in order to avoid negative influences on its stability. In the future, only controlled clinical trials of approved cleaning regimes may yield valuable evidence as to whether a particular cleaning method contributes significantly to peri-implant tissue health.

## 5. Conclusions

Based on the results of the present study, the use of planimetric analyses of SEM images is suitable for assessing the projected surface contamination of custom abutments. Upon delivery, monotype abutments fabricated in a CAD/CAM center of an implant manufacturer showed a significantly higher level of production-related contamination compared to hybrid abutments processed in a dental laboratory. In contrast to hybrid abutments, which have not shown a significant cleaning effect with the applied ultrasound protocol, the degree of contamination caused by the computer-aided fabrication of CAD/CAM monotype abutments can be significantly minimized using a three-stage ultrasound protocol.

## Figures and Tables

**Table 1:** Type, material, manufacturer, and sample size of tested CAD/CAM abutments.

Group	Abutment Type	Material	Product & Manufacturer	No. of Samples
**A**	Monotype Abutment	Titanium Grade 5	CADAbut F, CAD Titanium, Semados SCX, BEGO Implant Systems	7
**B**	Monotype Abutment	Y-TZP Zirconia	CADAbut F, BeCe CAD Zirconia XH, Semados SCX, BEGO Implant Systems	7
**C**	Hybrid Abutment	Y-TZP Zirconia Coping/Ti-Base	CADAbut D, Zirconia LT CAD on Ti-Base, Semados SCX, BEGO Implant Systems	7
**D**	Hybrid Abutment	Lithium Disilicate Coping/Ti-Base	CADAbut D, IPS e.max CAD LT/ Ivoclar Vivadent AG on Ti-Base, Semados SCX, BEGO Implant Systems	7

**Table 2:** Chemical analysis of particulate residues on IPSemax/Ti-Base samples before ultrasonic cleaning by element and maximum/minimum atomic concentration.

Element Number	Element Symbol	Element Name	Atomic Conc. (%) Max./Min.
8	O	Oxygen	74.63–60.21
14	Si	Silicium	28.71–0.92
40	Zr	Zirconium	19.66–1.01
19	K	Potassium	9.19–0.58
56	Ba	Barium	5. 97–0.79
13	Al	Aluminum	4.25–0.58
20	Ca	Calcium	3.54–0.00
11	Na	Sodium	2.84–1.64
39	Y	Yttrium	2.24–0.00
16	S	Sulfur	1.53–0.00
12	Mg	Magnesium	1.52–0.00
30	Zn	Zinc	0.91–0.00
15	P	Phosphorus	0.72–0.00

**Table 3:** Chemical analysis of particulate residues on zirconia/Ti-Base samples before ultrasonic cleaning by element and maximum/minimum atomic concentration.

Element Number	Element Symbol	Element Name	Atomic Conc. % Max./Min.
6	C	Carbon	80.54–44.52
8	O	Oxygen	30.84–9.24
7	N	Nitrogen	20.84–0.00
40	Zr	Zirconium	12.59–3.81
39	Y	Yttrium	9.18–1.05
22	Ti	Titanium	4.50–0.00
14	Si	Silicium	1.60–0.00
13	Al	Aluminum	1.11–0.00

**Table 4:** Chemical analysis of particulate residues on monotype titanium samples before ultrasonic cleaning by element and maximum/minimum atomic concentration.

Element Number	Element Symbol	Element Name	Atomic Conc. (%) Max./Min.
22	Ti	Titanium	80.70–28.36
6	C	Carbon	65.06–31.94
40	Zr	Zirconium	31.95–0.00
13	Al	Aluminum	14.61–3.42
11	Na	Sodium	5.04–0.00
39	Y	Yttrium	3.55–0.00
23	V	Vanadium	2.60–1.15
20	Ca	Calcium	2.40–2.01
16	S	Sulfur	1.14–0.00

**Table 5:** Chemical analysis of particulate residues on monotype zirconia samples before ultrasonic cleaning by element and maximum/minimum atomic concentration.

Element Number	Element Symbol	Element Name	Atomic Conc. (%) Max./Min.
6	C	Carbon	97.29–0.00
8	O	Oxygen	64.25–43.37
40	Zr	Zirconium	50.84–32.26
39	Y	Yttrium	5.79–3.49

**Table 6:** Descriptive analysis; box plot of projected surface contamination of all abutment samples before and after cleaning in pixels and percent.

Descriptive Analysis						
	N	Mean	Median	Min.	Max.	St. Dev.
Pixel total before cleaning	28	459,105.0	468,155.0	327,680.0	515,278.0	42,943.82
Pixel of contaminants before cleaning	28	11,631.4	2347.0	28.0	116,728.0	23,133.89
% Contamination before cleaning	28	2.4	0.5	0.0	24.6	4.89
Pixel total after cleaning	28	437,911.2	436,259.0	388,941.0	502,181.0	32,825.33
Pixel of contaminants after cleaning	28	537.1	360.5	26.0	1875.0	568.20
% Contamination after cleaning	28	0.1	0.1	0.0	0.4	0.12
% Difference before/after	28	2.3	0.3	-0.1	24.6	4.88
box plot of projected surface contamination of all abutment samples before and after cleaning in pixels and percent	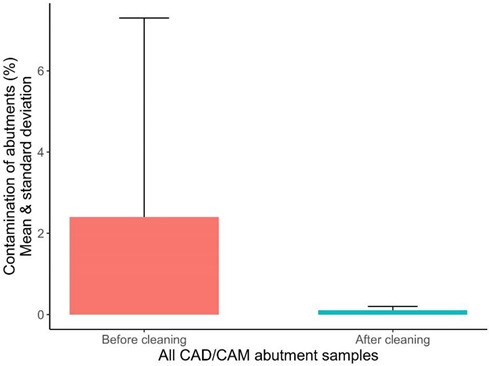					

**Table 7:** Comparison of projected surface contamination between monotype and hybrid abutments before and after cleaning in percent; Mann–Whitney U-Test; box-plot graphic.

**Monotype Abutments: Titanium/Zirconia**						
	**N**	**Mean**	**Median**	**Min.**	**Max.**	**St. Dev.**
% Contamination before cleaning	14	4.861380	2.899032	0.900291	24.61993	6.098782
% Contamination after cleaning	14	0.192341	0.149575	0.025887	0.42001	0.136643
% Difference before/after	14	4.669040	2.741231	0.532721	24.59404	6.141301
**Hybrid-Abutments: IPSemax-/Zirconia and Ti-Base**						
	**N**	**Mean**	**Median**	**Min.**	**Max.**	**St. Dev.**
% Contamination before cleaning	14	0.030569	0.012648	0.006283	0.097961	0.030976
% Contamination after cleaning	14	0.050087	0.030263	0.005599	0.147662	0.044470
% Difference before/after	14	−0.019518	−0.008203	−0.109325	0.028793	0.043838
**Mann–Whitney U-Test**						
	**Rank Sum**	**Rank Sum**	**U**	**N**	**N**	***p*-value**
	**Monotype**	**Hybrid**		**Monotype**	**Hybrid**	
% Contamination before	301.0000	105.0000	0.00000	14	14	0.000000
% Contamination after	276.0000	130.0000	25.00000	14	14	0.000421
% Difference before/after	301.0000	105.0000	0.00000	14	14	0.000000
box-plot graphic	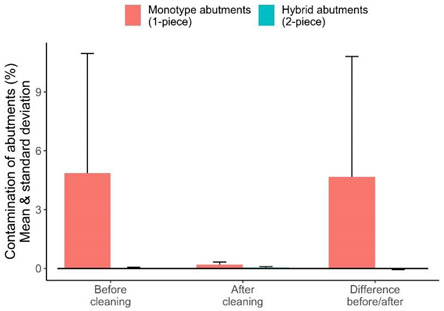					

**Table 8:** Projected surface contamination of titanium and zirconia monotype abutment samples before and after cleaning in percent; mean, median, min., and max. Total contamination change in percent; box-plot graphic.

	**N**	**Mean**	**Median**	**Min.**	**Max.**	**St. Dev.**
**Titanium Monotype**						
% Contamination before cleaning	7	4.020973	2.924533	1.573535	8.789632	2.609841
% Contamination after cleaning	7	0.165439	0.111186	0.053941	0.379537	0.109002
% Difference before/after	7	−3.86	−2.82	−8.60	−1.47	2.55
% Total contamination change	7	−95.4	−96.5	−97.8	−92.6	2.2
**Zirconia Monotype**						
% Contamination before cleaning	7	5.701788	2.251034	0.900291	24.61993	8.492950
% Contamination after cleaning	7	0.219242	0.187964	0.025887	0.42001	0.163965
% Difference before/after	7	−5.48	−2.06	−24.59	−0.53	8.58
% Total contamination change	7	−87.1	−89.5	−99.9	−57.5	14.3
	**N**	**Mean**	**Median**	**Min.**	**Max.**	**St. Dev.**
**Titanium and Zirconia Monotype**						
% Difference before/after	14	−4.67	−2.74	−24.59	−0.53	6.14
% Total contamination change	14	−91.2	−93.5	−99.9	−57.5	10.7
box-plot graphic.	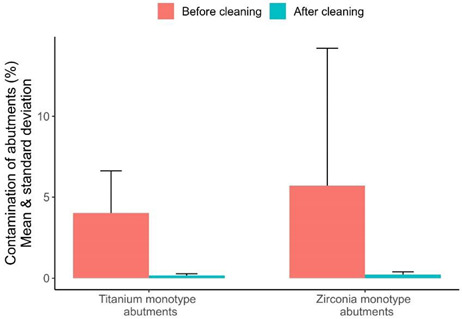					

**Table 9:** Projected surface contamination of hybrid abutment samples (IPSemax and Ti-Base, Zirconia and Ti-Base) before and after cleaning in percent; mean, median, min. and max. total contamination change in percent; and box-plot graphic.

	**N**	**Mean**	**Median**	**Min.**	**Max.**	**St. Dev.**
**IPSemax and Ti-Base**						
% Contamination before cleaning	7	0.013165	0.007598	0.006283	0.040418	0.012325
% Contamination after cleaning	7	0.017373	0.015093	0.005599	0.031881	0.008056
% Difference before/after	7	0.004	0.008	−0.020	0.023	0.013
% Total contamination change	7	82.6	104.3	−48.5	252.4	110.4
**Zirconia and Ti-Base**						
% Contamination before cleaning	7	0.047973	0.050000	0.010228	0.097961	0.034933
% Contamination after cleaning	7	0.082802	0.071295	0.028646	0.147662	0.041504
% Difference before/after	7	0.035	0.023	−0.029	0.109	0.059
% Total contamination change	7	286.6	97.3	−42.7	1007.6	443.2
	**N**	**Mean**	**Median**	**Min.**	**Max.**	**St. Dev.**
**IPSemax/Zirconia and Ti-Base**						
% Difference before/after	14	0.020	0.008	−0.029	0.109	0.044
% Total contamination change	14	184.6	100.8	−48.5	1007.6	327.8
box-plot graphic